# Let’s get it started: Eye Tracking in VR with the Pupil Labs Eye Tracking Add-On for the HTC Vive

**DOI:** 10.16910/jemr.15.3.10

**Published:** 2023-06-19

**Authors:** Judith Josupeit

**Affiliations:** Technische Universität Dresden, Germany

**Keywords:** Eye movement, eye tracking, virtual reality, HMD, HTC Vive, Pupil Labs, Unity, good practice

## Abstract

Combining eye tracking and virtual reality (VR) is a promising approach to tackle various applied research questions. As this approach is relatively new, routines are not established yet and the first steps can be full of potential pitfalls. The present paper gives a practice example to lower the boundaries for getting started. More specifically, I focus on an affordable add-on technology, the Pupil Labs eye tracking add-on for the HTC Vive. As add-on technology with all relevant source code available on GitHub, a high degree of freedom in preprocessing, visualizing, and analyzing eye tracking data in VR can be achieved. At the same time, some extra preparatory steps for the setup of hardware and software are necessary. Therefore, specifics of eye tracking in VR from unboxing, software integration, and procedures to analyzing the data and maintaining the hardware will be addressed. The Pupil Labs eye tracking add-on for the HTC Vive represents a highly transparent approach to existing alternatives. Characteristics of eye tracking in VR in contrast to other headmounded and remote eye trackers applied in the physical world will be discussed. In conclusion, the paper contributes to the idea of open science in two ways: First, by making the necessary routines transparent and therefore reproducible. Second, by stressing the benefits of using open source software.

## Introduction

In the past decades, virtual reality (VR) made its way from an abstract science fiction concept to easily implementable affordable consumer electronics. To allow the reader to experience the same progression from abstract to concrete, I will start with some general information about VR and eye tracking. Progressing with presenting easily applicable good practices for eye tracking in VR with the HTC Vive and the Pupil Labs eye tracking add-on throughout this paper.

In VR a three-dimensional computer-generated simulated scene is used to create a sense of “being there” sometimes called (tele-)presence (9; 59). Although different VR technologies co-exist, this paper focuses on head-mounted displays (HMD), also named VR-goggles. To create an experience of virtual realness, a variety of hard- and software components can be used that facilitate the immersion. As standard stereoscopic images are applied to allow depth perception via binocular disparities. Moreover, six degrees of freedom head-based rendering takes the users’ translational and rotational head movement into account. As an add-on manipulability of and interaction with the simulated objects can be achieved when controllers are used.

Especially for applied research, the use of VR comes with many advantages: VR allows for controllable environments and the reproducibility of particular settings. This also implies that cover stories are relatively easy to implement. Thus, the immersive visual stimuli do facilitate credibility. The possibilities of displaying visual stimuli range from the resemblance of real physical objects to complete artificial ones. Via logic components of the application, physics can be applied that as a default matches the empirical knowledge of the physical world but can also be diametrically opposed. In either case, these simulations allow studying the respective physiological reactions of the autonomous nervous system in a highly controlled but simultaneously immersive setting. In addition, participants seem to be more motivated and willing to adhere to the procedure compared to classical laboratory experiments (3), as participants have reported more fun and enjoyment, or engagement and motivation compared to non-immersive setups (11; 36).

The use of VR generates accessible metadata, e.g. head movement data required for head-based rendering can be easily recorded. In addition to the rotational and translational metadata, that can be accessed through the build-in sensors of the HMD and its motion-tracked controllers, eye tracking in VR is applied in many areas of cognitive research: For instance focusing on spatial orientation (33), medical training (35) or marketing (31). Combining spatial and temporal eye data allows for the allocation of eye movement events such as fixations (i.e. gaze is relatively stationary) and saccades (i.e. eye movements) (54). In combination with additional spatial and temporal information about the displayed events, scan paths can be derived and aggregated to heat maps. If available, predefined areas of interest (AOI) provide further process indicators e.g. dwell count and dwell duration (44).

Various suppliers for eye tracking solutions in VR exist. There are expensive HMD solutions that are manufactured for eye tracking in the workplace. Manufacturers promise more efficient prototyping, training, and research (66). Other HMDs with an eye tracker as a standard feature belong more to consumer electronics (e.g. 25). The disadvantage of most of these solutions is the black box algorithm that processes the eye tracking data. Depending on the software used to actuate the eye tracker, only aggregated data is available limiting the research questions that can be addressed (61).

In contrast, open source solutions might be an affordable, transparent, and flexible alternative. One of these eye tracking systems will be discussed in this paper: The Pupil Labs eye tracking add-on for the HTC Vive (47): a binocular add-on solution with a maximum sampling rate of 200Hz. According to the manufacturer, the gaze accuracy is about 1.0°, the gaze precision is about 0.08°, the camera has a latency of 8.5 ms, and the processing latency is 3 to 4 ms depending on the CPU (Core i5).

However, such an affordable solution requires additional effort in the setup. To facilitate the start with eye tracking in VR, I would like to focus on practical aspects of implementing eye tracking in VR, which have not been mentioned elsewhere. A detailed overview together with a case study with the same hardware is provided by Clay et al. (14). In case the reader wants to get informed about publications and projects from diverse research fields that applied Pupil Labs eye trackers in VR I recommend taking a look at the manufacturer’s publication list filtering for VR (48). Even for those who are already familiar with the technology, the present paper might contain some helpful practical suggestions. Novices in this field will be guided through the overall process and pointed to potential pitfalls and risks.

I will go through the necessary steps in chronological order: First, I will focus on the mounting of the hardware, then continue with software integration of the Pupil Core apps and Unity, as well as suggestions for experimental procedures, accessing raw data and preprocessing, and conclude with maintenance recommendations for large-scale laboratory studies.

## Mounting

Starting with the unboxing of the Pupil Labs eye tracking add-on, the package includes two distinct hardware components. The first component is the eye tracker − the cameras and infrared illuminators − installed on clip-on attachment rings, one ring for each lens of the HMD. The second component is a USB-C to USB-A cable that powers the lenses and streams the data. Compared to “plug-and-play” solutions an extra step is needed, which is mounting the eye tracker onto the lenses of the HTC Vive.

In general, the mounting is well documented and helpful instruction videos are provided on Pupil Labs’ YouTube channel (45). Nevertheless, the instructional video for the HTC Vive should be more precise since the attachment rings are quite delicate and prone to damage. There is a comment under the instructional video on YouTube that suggests turning the knob of the HTC Vive on the lower right side that adjusts the interpupillary distance (IPD) to the maximum to avoid obstruction by the HMD’s plastic facerest when mounting the eye tracker. However, in my opinion, the HMD’s facerest still obstructs access to the lenses, if it is not removed. Therefore, disassembling all parts that are interfering seems appropriate^1^.

**Figure 1:**
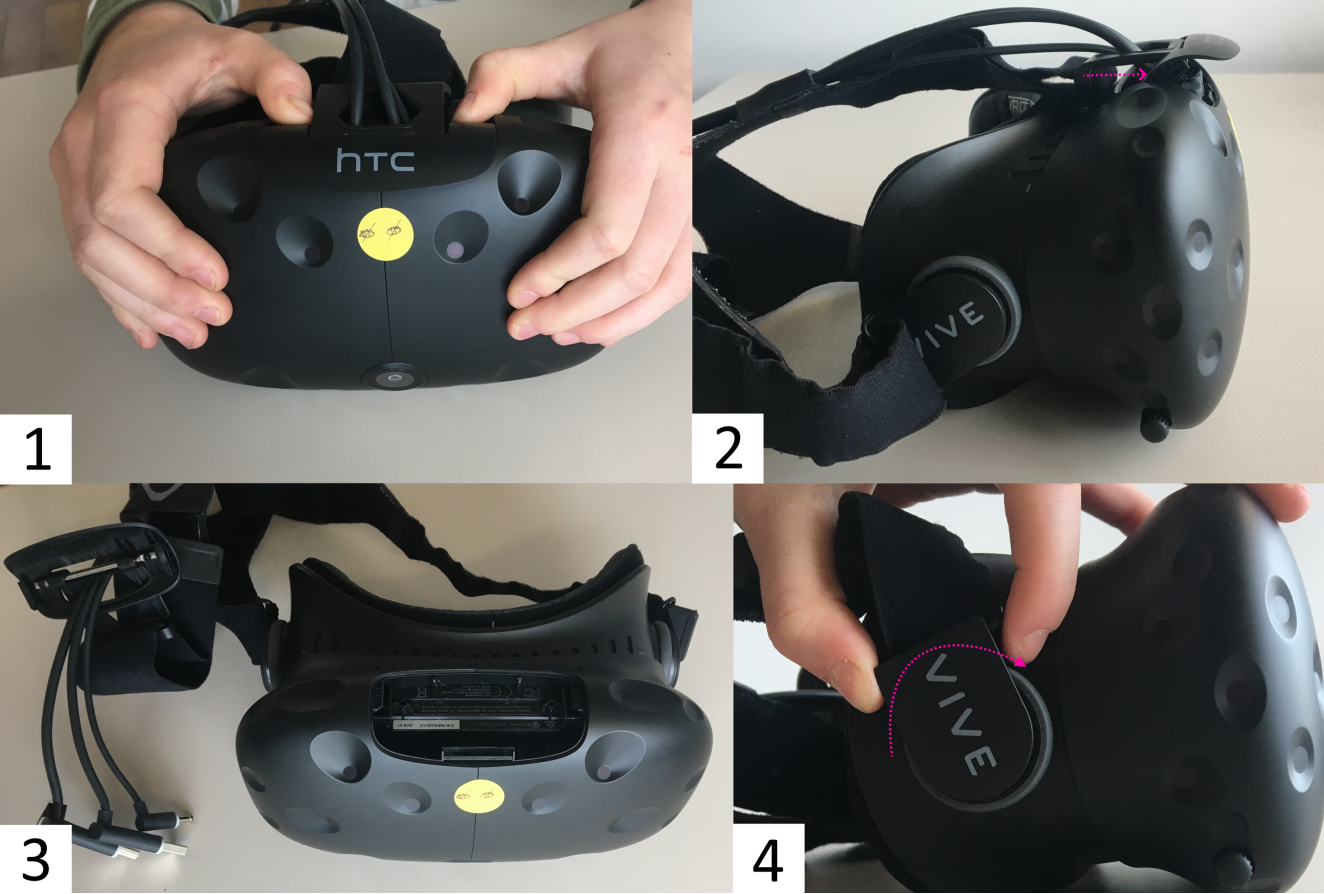
Steps Necessary to Remove the Cables and Head Straps of the HTC Vive Note. 1.1 and 1.2. Removing the cover of the connector cables, 1.3 Connector cables unplugged, 1.4 Removing the head strap mount by turning it upwards.

**Figure 2:**
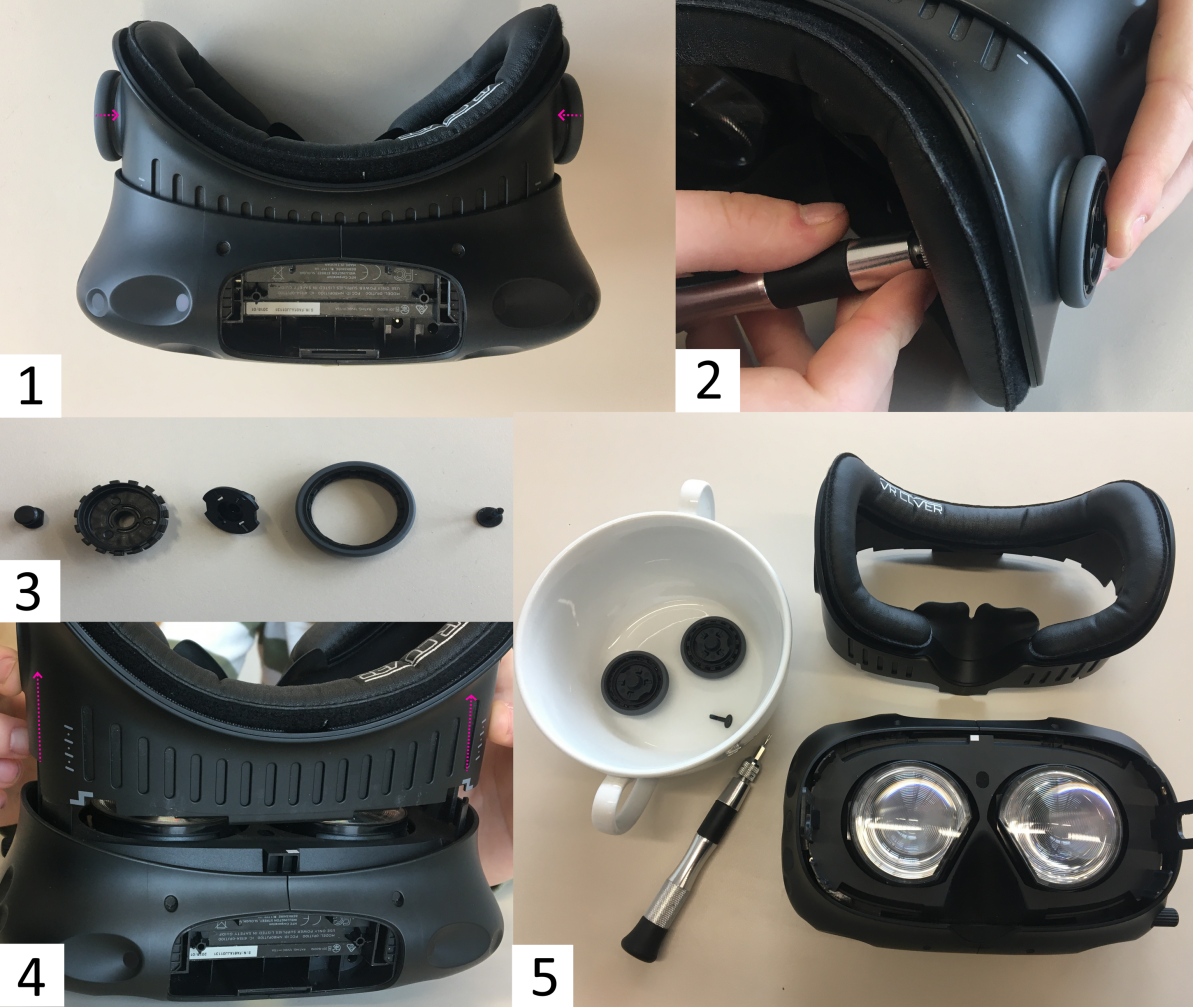
Steps Necessary to Remove the Eye Relief Adjustment Mechanism and the Facerest of the HTC Vive Note. 2.1. Locking the eye relief adjustment mechanism 2.2 Loosen the screws of the mechanism with a Torx T6 screwdriver 2.3 The five parts of the eye relief adjustment mechanism 2.4 Removing the HMD’s facerest 2.5 Result of dismounting the HMD.

Before the facerest can be removed, the cables, the elastic head straps, and the eye relief adjustment mechanism need to be disassembled (see Figures 1 and 2). Start with removing the connector cables, which are at the top of the HMD (see Figures 1.1 to 1.3). This part is covered with a plastic locking bolt that can be removed by pushing each corner of the cover down and sliding it toward the front of the HMD. Under the cover are the connector cables (HDMI, USB-A 3.0, and DC barrel jack for power supply, sometimes an additional 3.5 mm audio jack). Unplug all cables. Put the cables and the cover aside. Then the mount of the elastic head straps on either side of the HMD, which sits on top of a grey ring that is part of the eye relief adjustment mechanism, can be released. For this purpose simply turn the plastic bracket upwards, until you hear it click, which indicates it is loose (see Figure 1.4). Do apply only gentle force because the dents of the bayonet mount are made of thin plastic.

Now you can put the head straps aside and continue with removing the facerest of the HMD (see Figure 2). Make sure that the mechanism for the adjustment of the eye relief is locked, which means that the grey rings are pushed down on either side of the HMD’s facerest (see Figure 2.1). Next, you can loosen the mechanical fastening of the cogs that adjust the eye relief. To do this, use a Torx T6 from inside the HMD (see Figure 2.2). Once the screws are loose, the mechanism will come apart in five parts the grey ring, the cog, the socket, the nut, and the screw. Memorize the colocation of the parts of the mechanism (see Figure 2.3). In general, for (de-)assembling it is recommended to use a container for all loose parts. Finally, pull away the facerest of the HMD (see Figure 2.4). As a result, you will gain unobstructed access to the lenses (see Figure 2.5). Now, the attachment rings from the Pupil Labs add-on can be gently clipped onto the lenses of the HTC Vive. Align the bare flexible printed boards in parallel to the HMD’s facerest. Check its fit by cautiously putting the facerest back on, before you begin to re-assemble the HMD.

To this end, take the steps in reverse order. Let gravity help you when reassembling the eye relief adjustment mechanism, by pushing the HMD’s facerest to the minimum (see Figure 3.1). The nut of the eye relief adjustment mechanism has to line up precisely with the plastic socket, which itself needs to line up precisely with the slot of the facerest. Once all parts are back in place, fasten them. Avoid overtightening the screws and make sure that the eye relief adjustment is working. Enable the mechanism by pushing the grey rings outwards and turn them to check whether the eye-relief changes, i.e. the facerest is rolling out.

Next, pull off the face cover foam, pass the USB-C connector under (see Figure 3.2), and put the foam back on again. For laboratory studies as well as for public demonstrations a damp wipeable PU leather foam face cover with disposable single-use hygiene covers on top is recommended. If the cover has not been changed previously, this would be a good opportunity to do so.

Continue by closing the flexible head straps outside the HMD's facerest by turning them down. Thereafter, plug in all cables and close the cover of the head compartment again.

Now as the mounting is completed, the USB-C connector should be attached to the computer using the 1.5 m long USB-C to USB-A cable that came with the package. Use a USB-A 3.+ socket of this computer to enable a sufficient data transfer rate. The computer should not only record the eye tracking data but also render the VR environment. This enables initializing the recording of the eye tracker and saving the event file simultaneously. Moreover, synchronizing the timestamps that apply different time formats will be easier.

**Figure 3:**
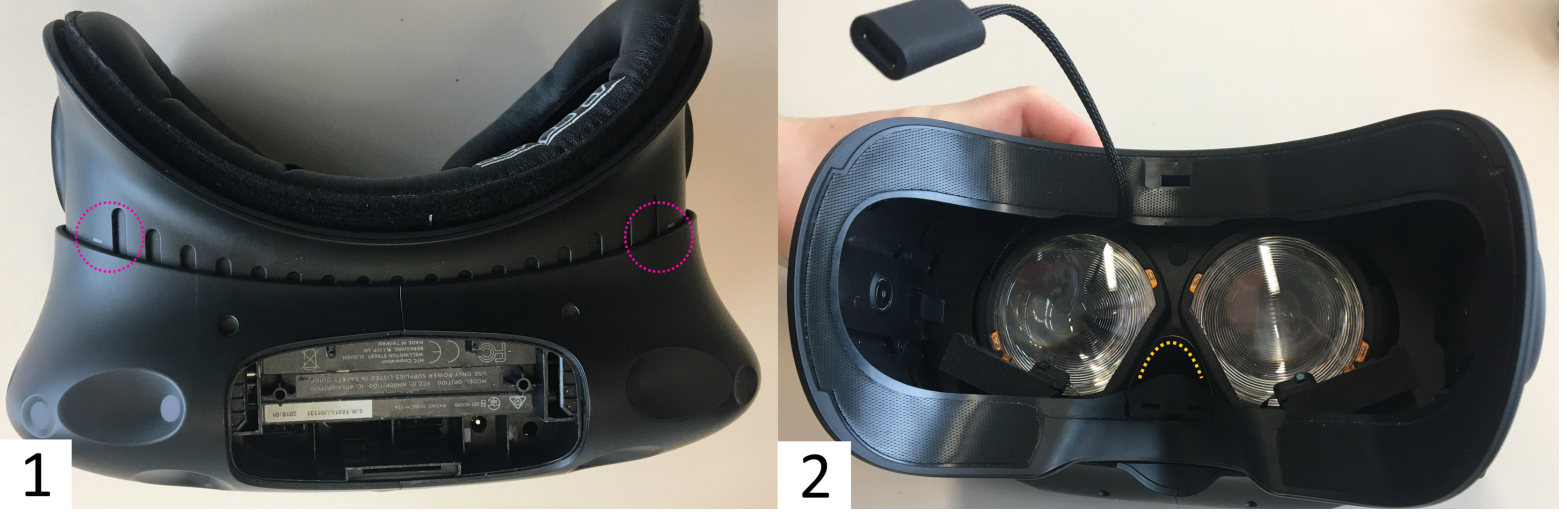
Precautions for Reassembling the HTC Vive with the Pupil Labs Eye Tracking Add-On Clipped onto the Lenses Note. 3.1. Facerest at the minimum position concerning eye relief 3.2. HMD with the add-on installed and face cover foam removed.

**Figure 4:**
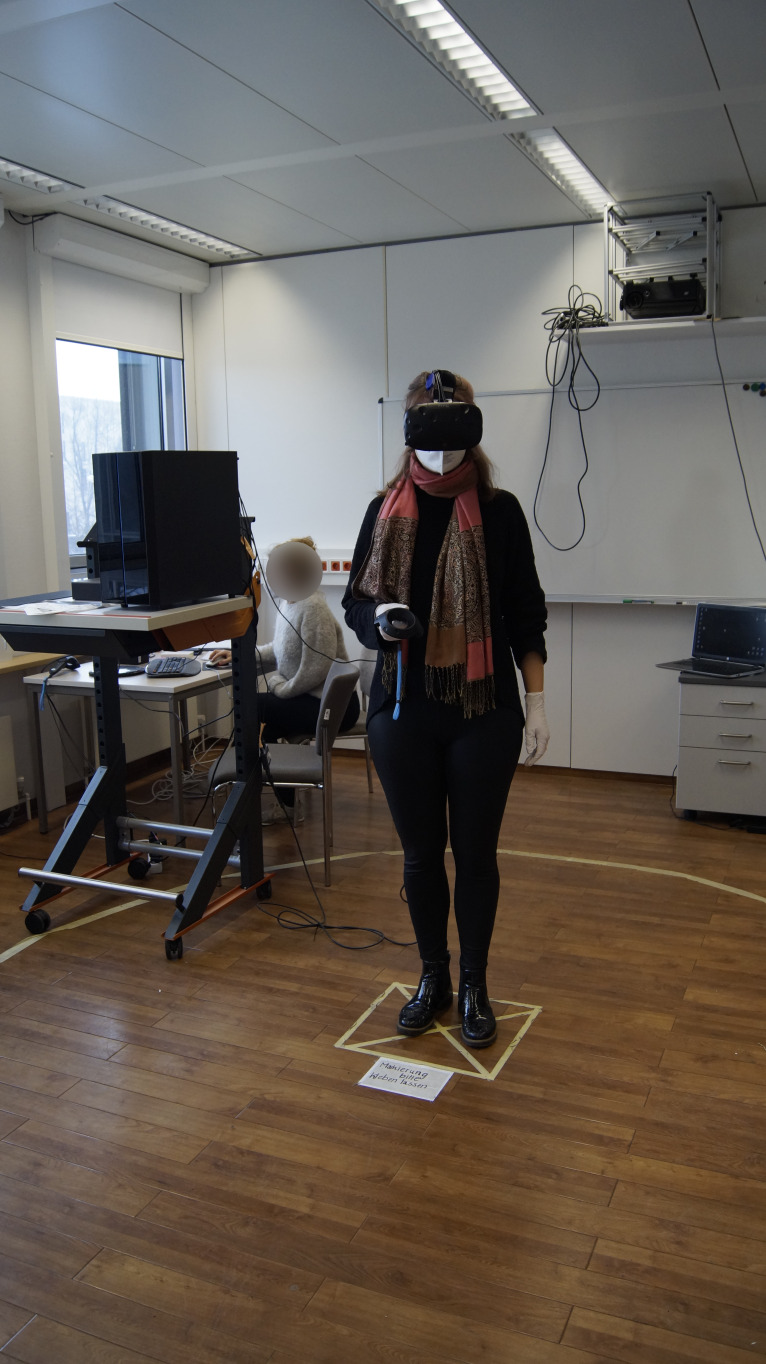
Re-staged Experimental Setup for Room-Scale VR Note. A re-staged experimental setup with the computer used for eye tracking and rendering on a wheeled lectern to enable adjustments for room-scale VR.

The 1.5 m long USB-C to USB-A cable should be used without any modification, which unfortunately limits the range of motion. Although not recommended by the supplier, modifications tried – an active USB-A extension cable, as well as connecting the cable to the spare USB-A slot of the head compartment of the HMD −, failed, potentially because of low voltage. To increase the range of motion, the computer can be mounted on something mobile and adjustable, such as a wheeled lectern (see Figure 4). Using a backpack computer might be another option, although this technology is discontinued, as it was received as overpriced and cumbersome (20; 26).

## Software Integration

In the upcoming paragraphs, different font styles will be used for more clarity: names for scripts and prompts will be written in Courier New, whereas names for prefabs and components will be written in *Italics*.

### Pupil Labs Core Apps

To get the latest version of the Pupil Labs Core apps the reader is referred to the Pupil Repository on GitHub (28; 49). The Pupil Labs Core apps include three apps: Pupil Capture, Pupil Player, and Pupil Service. Whereas Pupil Capture and Pupil Service are for recording, streaming, and calibration, Pupil Player enables visualizing, preprocessing, and exporting the data. Therefore, Pupil Player will be addressed later in the sections Accessing Raw Data and Preprocessing.

Pupil Capture and Pupil Service differ concerning the GUI: Pupil Service contains only the two eye cameras, while Pupil Capture contains an additional window of the world camera, i.e. the third camera that records the displayed scene. One way to enable the world camera view in Pupil Capture is to use a Screencast, which will be the focus of an upcoming section. Accordingly, Pupil Service requires less processing power, which is advantageous for higher sampling frequencies of the eye tracker. However, if one wishes to start the recording through the VR application, the use of Pupil Capture is indicated, as the *Recording* component is not supported by Pupil Service.

The first time you plug in the USB-A connector of the eye tracker into the computer the drivers for the eye cameras will be installed automatically. Make sure that both eye cameras are displayed on the screen when starting Pupil Capture or Pupil Service. When the HMD is worn already, it might happen that the eye camera for the right eye from the wearer’s point of view (camera 0) is not visible. This usually happens because the bare flexible printed circuit board underneath the nosepiece is disconnected when the HMD is sitting very tight on the nose. Moreover, if both eye cameras are not available, it might help to uninstall the drivers and plug the USB-A connector in again. If none of the usual troubleshooting measures work (e.g. restarting the computer or unplugging the USB-A connector), the problem may be due to a rupture in the flexible circuit board. The rupture will be obvious to you by a visual inspection. If you are technically inclined, you can solder a bridge as a makeshift solution. Other ideas to prevent cable rupture will be covered in the Maintenance section.

For the integration of the software, it is enough to know the differentiation and unique characteristics of the Pupil Core apps. I will refer to some basic settings of the Pupil Core apps in the section Recommendations for the Experimental Procedure.

### Unity

**Figure 5:**
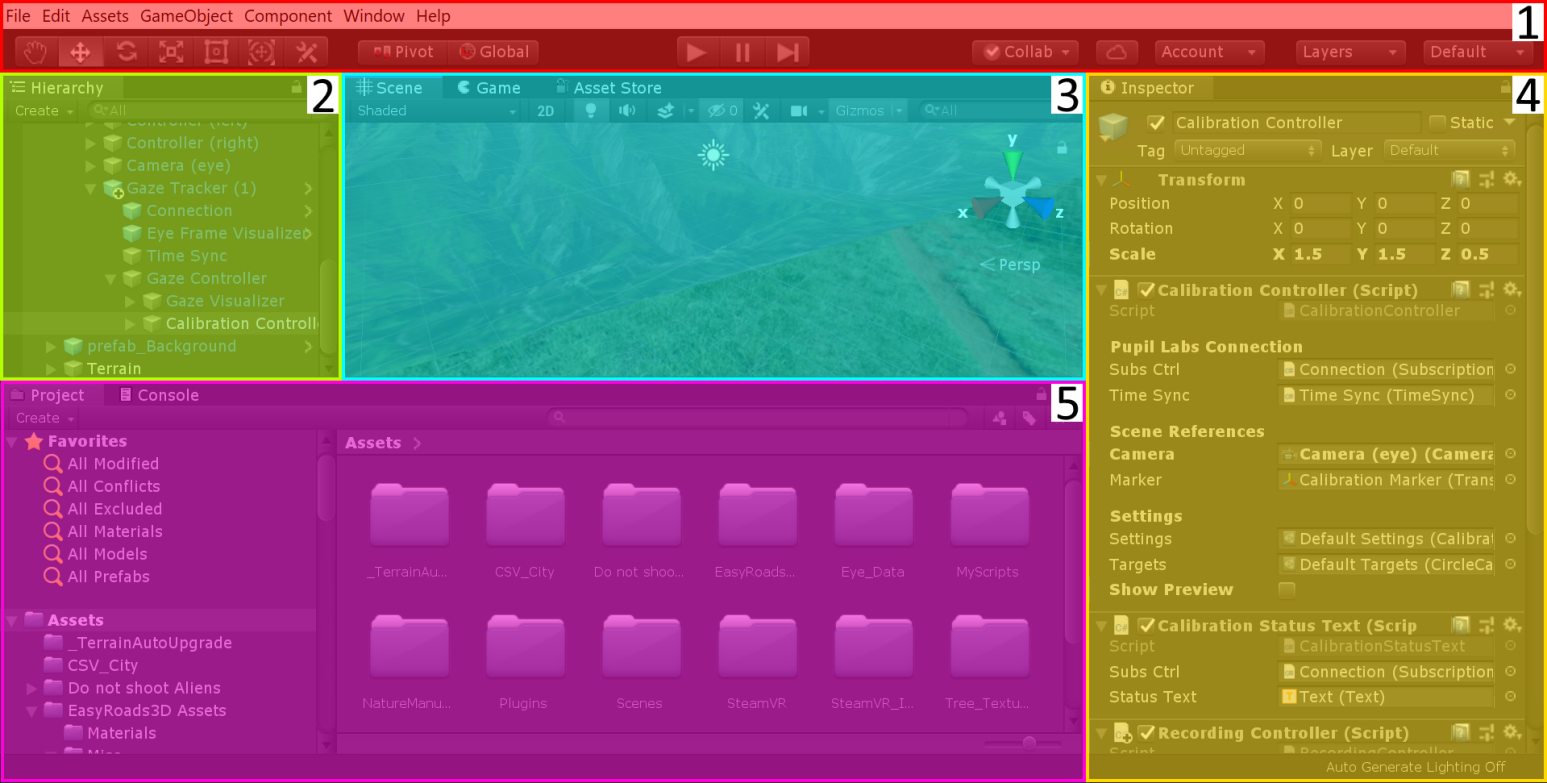
The Five Sections of the Default View of the GUI in Unity Note. 5.1. Toolbar 5.2 Hierarchy: currently the Calibration Controller is selected 5.3. Scene view is selected; the following tabs are the Game view and the Asset Store. 5.4. In the Inspector the adjustable settings of the Calibration Controller are shown. In case you wish to change the Settings or Targets just double-click on the ScriptableObject 5.5. The Project browser is displayed; the following tab is the Console.

The following paragraphs refer to Unity Professional (v 2019.1.1.1f1). The professional license is free of charge if you are working in academia for educational purposes. The default view of the GUI can be split into five sections (see Figure 5). First, there is *the toolbar* section for editing the current project by importing packages and components, adjusting settings, and compiling the scene for testing, as well as building the final application. The second section, the *Hierarchy*, contains all game objects in the scene. You can change the hierarchy of game objects by dragging and dropping game objects onto one another. The objects hierarchically lower are called *child objects* and depend in all degrees of freedom on the object they are attached to, i.e. the *parent* (game) *object*. Third, in the center of Unity’s GUI, there is the *Scene* view for previewing, the *Game* view for testing, and the *Asset Store* for downloading preconfigured assets. Many facilitating assets are available for free: For instance, the *SteamVR Plugin* helps with the integration of the HMD and its controllers, while other assets contain meshes and materials for 3D-game objects. Forth on the right side, the *Inspector* displays detailed information about the game object that is currently selected in the *Hierarchy*. In the *Inspector* the selected game object can be adjusted in size position and rotation. Moreover, scripts − written in C#. – and components − e.g. a *Rigidbody* for enabling Unity’s physics engine − can be attached to the game object. Finally, in the lower part of the GUI, there is the *Project* browser and *Console* tab. The *Project* browser shows all folders of the current project. If you want to add a game object to the current scene that is already imported into your *Project* folder just browse through the *Project* folders, drag and drop it into the *Hierarchy*. The *Console* is important during testing the Unity scene, i.e. when you press play in the *toolbar*, according to the settings warnings and, or error messages, and logged information are displayed.

In case the Unity scene does not define all game objects as *child objects* of the *main camera*, the virtual height impression depends on the physical height of the user. If you want to unify the camera perspective for users of all heights, add a *Character Controller* to the *main camera* component opened in the *Inspector*. The character controller can define a fixed virtual height. The importance of the y-axis constraint on the main camera depends on the VR setup: While it may be important for room-scale VR or a standing position with a perspective higher than the user's physical height, the y-axis constraint may be marginal for a seated VR (55). Nevertheless, there may be good reasons not to fix the perspective, such as ecological validity or a reduced immersion.

In addition, to the previously mentioned *Asset Store* some facilitating and beginner-friendly frameworks for designing VR experiments with Unity can be recommended (6; 10). In case you need some special 3D-game object, that is not available via the *Asset Store*, the free and open source software Blender might be an appropriate alternative (7). For creating 3D-game objects with Blender keep in mind, that Unity uses a left-handed y-up coordinate system, whereas Blender uses a right-handed z-up coordinate system.

The Pupil Labs Unity package *hmd.eyes* needed to actuate the eye tracker from inside the Unity application is available on GitHub (46). For general information refer to the Pupil Labs VR/AR developer docs that not only support the game engine Unity (2018.4+), but also the software development platform Vizard (Worldviz), which is not covered here. You can import the package via the import custom package prompt through the toolbar into your Unity project. In the following sections, I will focus on different components that are included in the package *hmd.eyes* and are more or less relevant when actuating the Pupil Labs eye tracker inside Unity. The package *hmd.eyes* requires the Pupil Core apps to be v 2.0+, which will be the case if you followed the instruction and installed the latest version.

If you just want to get started with eye tracking in VR, I recommend using the *Gaze Tracker* component from the package *hmd.eyes*. After importing the custom package into your Unity scene, you can attach the *Gaze Tracker* to your *Camera Rig* of the *main camera*, as a *child object* by dragging it into the *Hierarchy*.

#### Calibration

Regardless of the research question, calibration of the eye tracker is required to map pupil positions to corresponding gaze positions in the VR scene. For the calibration procedure, a neutral background with equal brightness compared to the VR scene is recommended to minimize visual distraction. I suggest using Blender to create a custom prefab like a tube that can be loaded into Unity as a game object. The tube can be displayed during the procedure and destroyed once the calibration was successful via a script.

When using the *Gaze Tracker* component keep the *Gaze Visualizer* (see Figure 6.1), which highlights with a sphere the Unity object that is currently fixated by the user, and the *Eye Frame Visualizer* (see Figure 6.2), i.e. a screencast of the eye cameras into the Unity application, disabled during the calibration. Open the *Calibration Controller* component in the inspector. In the Calibration Controller script add your *main camera* as Reference Camera and enable Show Preview which will display the targets used for the calibration procedure. You can change the Calibration Settings and Targets to your needs via changes in the *Inspector* (see Figure 5.4). Make sure to calibrate the whole field of view you wish to display stimuli in. For depth perception in VR, keep in mind that Unity units can be theoretically arbitrary, but are recommended to be thought of as meters as the physics settings assume so (62), e.g. gravity is set to the earth’s equivalent of $9.81\frac{m}{s²}$ (0, -9.81, 0).

Open the *Inspector* for the object *Canvas* which is a child object of *Status Text*. Select the *Screen Space – Camera* as the Render Mode. Assign the *Camera Rig* of your *main camera* as the Render Camera to display the respective text inside the HMD irrespective of the head position and rotation.

If you want to validate your calibration, add a second *Calibration Controller* as a child object to the *Gaze Tracker* and rename it to *Validation*. Use a different order of the displayed targets for your validation. Therefore, you need to adjust the Settings and Targets once again in the *Inspector*. Furthermore, open the C#-script and change the keycode for starting the Validation process from C to V.

Between the experimental sessions, allow the participants to rest without wearing the HMD, which reduces the likelihood of discomfort or visually induced motion sickness (VIMS). Begin each new VR application with a calibration. To control for the effect of slippage repeat the calibration at the end of each session; therefore just initialize the calibration process a second time by pressing C. If your environment is causing some form of VIMS, a recalibration may be weighed against reducing the overall exposure time (53).

#### Visualizing Scan-Paths and Areas of Interest

If it is of interest, which objects a participant is fixating on, the *Gaze Visualizer* needs to be enabled after the calibration process and the Gaze Origin should be set to the *Transform* of the *main camera*. Furthermore, the game objects of interest should have *colliders*. *Colliders* are invisible meshes which mimic the shape of the game object and are needed by Unity’s physics engine to enable interaction with game objects. Otherwise, the *ray cast* of the *Gaze Tracker* cannot hit them. You could think of a *ray cast* as a laser beam that starts from a fixed point (origin) and radiates on all *colliders* that are in alignment (direction and distance).

The visualization of the gaze can be enabled with the *Gaze Visualizer* component of the *Gaze Tracker*. In general, this is often distracting but can be helpful when using gaze-based interaction or when simulating visual impairments like a cataract (34). For these purposes, the *Gaze Visualizer* should be enabled and modified by adding either a script or changing the Materials of the Sphere Cast. In case the *ray cast* is too coarse, modify the scripts for the Confidence Threshold and the Sphere Cast Radius according to your needs through the sliders in the *Inspector*.

To obtain scan paths for post-processing, the hits of the *Gaze Visualizer’s* *ray cast* need to be saved. Moreover, for AOI you should label your *game objects* of interest by using custom tags or for the areas of interest of large game objects by adding specific *Mesh colliders* as *child objects*.

#### Recording

For the synchronization between the system time in Unix epoc and Pupil Labs time in arbitrary timestamps, it is recommended to start the recording of the Pupil Core app from inside the Unity application. This also facilitates the handling of the software, once the Unity application is build. Add the Recording Controller script to the *Gaze Tracker* component opened in the *Inspector* to enable the opportunity to start the recording from inside the same application. Select the Connection (i.e. *Request Controller*) as the Request Ctrl*.* Allow the control Start Recording for starting the recording from inside the Unity application by pressing R on the keyboard, but do not enable the Stop Recording control to reduce the likelihood of experimenter’s error by pressing R multiple times.

The recording will start on Update(), meaning the script will be called once per frame and the recording will start in the respective frame when the key is pressed on the keyboard (63). When using a custom path for saving the recordings, the path should be identical to the path for the Unity event file for higher clarity. As long as the Unity Editor is used, using the path to the project folder is fine. Keep in mind that the Unity project folder is normally not the folder you wish to build your Unity application in.

#### Screencast

In some cases, you might want to include a screencast, i.e. recording the virtually displayed scene, to illustrate the functionalities of the eye tracker or for troubleshooting. Make sure that the screen capture software records with the appropriate frames per second for a jitter-free screen recording (for a screencast with OBS see OBS, 42).

To achieve a screencast with sufficient frames per second, but a limited resolution, attach the Screen Cast script to the *Gaze Tracker*. In the *Inspector*, assign the *Screen Cast Camera* as the Centered Camera. Use the respective Request Ctrl as C*onnection* and the Time Sync as *Time Sync*. Now the *Game* view will be displayed in the world video window when you are using the Pupil Core apps Pupil Capture or Pupil Player (for replay). The screencast will be saved as an additional file in the same folder as the other recording information. If the Unity scene is visually complex, the Clipping Planes of the *main camera* and renderings of the *Skybox* may look slightly different in the world video compared to Unity’s *Game view*.

Moreover, you might want to visualize the functionality of the *Gaze Tracker* for a demonstration or documentation*,* to this end enable the *Gaze Visualizer* (see Figure 6.1). If you additionally want to include the eye images of the eye tracker inside the Unity application, the *Eye Frame Visualizer* of the *Gaze Tracker* component needs to be enabled (see Figure 6.2). However, due to the demanded processing power, a jittered rendering can result and sampling frequency might become irregular. Thus, a screencast is generally neither recommended nor needed for most research questions.

#### Saving Unity Events

To get inspiration for structuring and writing an event file in C#, the reader is referred to the Electronic Supplementary Material section. In general information about the head movements of the participant, the use of the controller, and what was displayed at a particular time, needs to be saved in an additional file with a code for all planned or initialized Unity events. This can be achieved by creating a new C#-script attached to the Unity scene. It allows saving a csv-file including the temporal, spatial, and virtually displayed information.

The sampling frequency will be depending on the frame rate when the script is called through the event function Update(). This event fiction is applied for all *Gaze Tracker* functionalities triggered inside Unity. As this function is depending on the volatile frame rate, it might result in variable intervals of records. Nevertheless, there is no benefit in rendering faster than the display refresh rate of 90 Hz. Thus sampling of the Unity event file around 60 to 120 Hz is common practice (6). Additionally, using Unity timestamps based on the system time in milliseconds is recommended (for Windows see Microsoft, 38).

**Figure 6:**
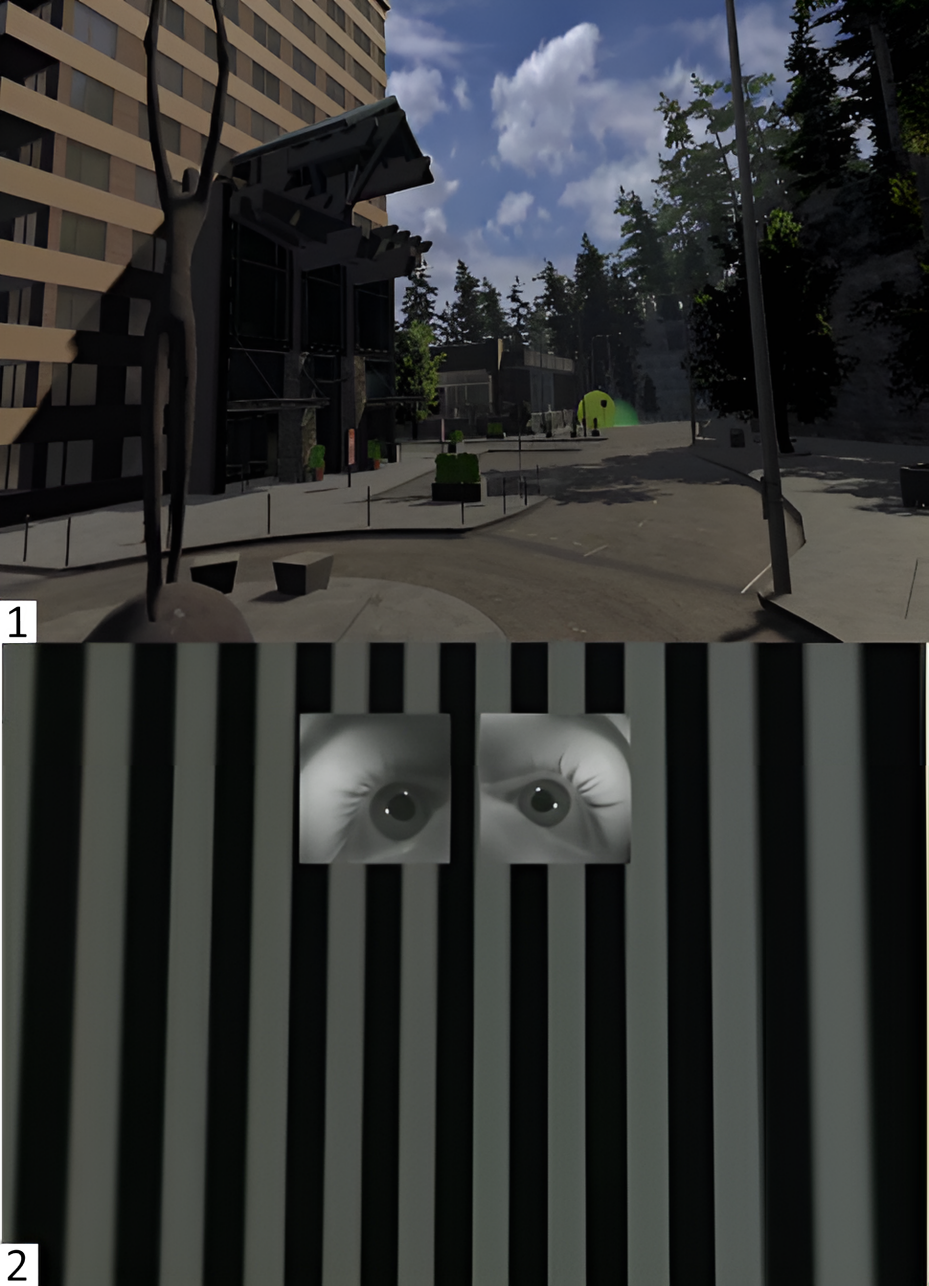
Demonstration of the Gaze Visualizer and Camera Images of the Eye Frame Visualizer Note. 6.1. The ray cast of Gaze Tracker illustrated by a yellow sphere (Gaze Visualizer) is hitting an object of interest, the green glowing semi-circle in a complex VR environment 6.2. Displayed images of the Eye Frame Visualizer for demonstrational purposes in a simple VR environment.

The event file can be labeled by using an *input field* as a GUI element to enter the participant code. The use of C, P, R, and/or V should be avoided as these key codes are already assigned to controls and would lead to an implausible event count. After entering the participant’s code and pressing the confirmation key, e.g. Enter on the numb pad, the saving script should be initialized. As a path for saving use Application.dataPath + “/folder_name/”. Additionally, use the system time of the initialization of the Unity application in the label. This adds redundancy to the file and helps to reduce the likelihood of experimenter’s error by mislabeling. Furthermore, it is recommended to sample the reference coordinates of the *main camera* *Position* and *Transform* in Unity so you can keep track of the head movement. This is important information for eye tracking in VR due to two reasons. First, as mentioned in the introduction rendering in VR is head-based. So, the position of the head needs to be known, including the content and the perspective at a time point, to visualize scan paths and heat maps. Second, there would be a chance of misinterpreting the vestibulo-ocular reflex, when head movement is disregarded. The vestibulo-ocular reflex is a reflex that stabilizes the line of sight during head motion via an eye movement in opposite direction to the head rotation (65).

In case you are using controllers with predefined or custom (key-)bindings, the controller input needs to be tracked, as either the events are triggered via the controller input or the controllers are applied for locomotion. In the former case, game objects and thus the visual input is changed. Therefore, you would additionally save the coordinates of the interactive game object. In the latter case, you will need to know the controller input to keep track of the changes in perspective that are not caused by head movement.

For scan paths and heat maps, the *ray cast hit marker* of the *Gaze Visualizer* in *Position* and *Transform* in Unity coordinates needs to be added to the event file. When you have predefined AOI, add each tag of the respective *collider* as a string to the event file and count the hits of the *ray cast* accordingly.

Furthermore, the timestamp when R is pressed i.e. when starting the recording in Pupil Capture needs to be logged. Additionally, use counters for the calibration and validation onset, the start of the VR application after successful calibration and validation, i.e. when the neutral background game object is destroyed, and for the end of the VR exposure if a re-calibration is applied. Moreover, use a counter for the onset of the planned events, especially when they are triggered irrespective of time spend in VR this is needed to match the eye tracking data with the Unity events.

#### Building the Unity Application

Before starting to build the Unity application try to find and delete all unnecessary code and game objects. I recommend using a checklist for a code review (12) if no experienced developers can provide support. Once you have your Unity scenes cleaned up and ready for the build, you need to make sure that the player configurations in the build settings have the *Scripting Runtime Version* set to *.NET 4.x Equivalent,* whereas the *API Compatibility Level* should be *.NET 4.x*. Enable the *Virtual Reality Supported OpenVR Virtual Reality SKD*. Moreover, to have easier handling between the VR application you are about to build and the Pupil Core apps set *Resolution* to *Windowed*. For more clarity build your application in an empty folder, as the Pupil Labs recordings and the Unity event file will be stored at Application.dataPath + “/”. Keep in mind that you have to change the path to this folder in the *Recording Controller* component opened in the *Inspector.*

## Recommendations for the Experimental Procedure

Further aspects that need to be considered in the context of planning the experimental procedure, are either applicable for eye tracking in VR in general or soft- and/or hardware specific for the Pupil Labs eye tracking add-on for the HTC Vive.

### Participants Inclusion Criteria

Not to mention that normal or corrected to normal vision is mandatory for experiments in VR and specifically in combination with eye tracking. If participants do need visual correction, they should wear contact lenses, but not glasses (57). This information should be included in the promotional e-mail or flyers used for recruiting. Additionally, the study information should include that participants should not wear eye makeup. Concerning eye makeup, keep some more permanent face modifications in mind e.g. permanent makeup or fake lashes. Furthermore, one can think of other exclusion criteria for VR studies such as migraineurs, pregnancy, and/or photosensitive epilepsy (for justifications see: 27; 37). Moreover, experiments working with VR should monitor VIMS during the VR exposure using self-rating scales. Three categories of self-rating-scales can be distinguished: Screening questionnaires which can be applied before the VR exposure to exclude highly susceptible participants (19), symptom questionnaires that illustrate the palette of VIMS symptoms (29; 32) and single-item questionnaires for a quick query throughout the VR exposure.(8; 30). Even if one is not interested in studying VIMS the participants’ safety needs to be ensured at all times, which is why the repeated use of a single-item questionnaire and monitoring of the participant through careful observation is indicated.

Since participants do not always know their visual acuity or what is meant by normal vision, two important prerequisites should be assessed before the VR exposure. First, visual acuity should be tested e.g. with the FrACT (4; 5). As the eye relief of the HMD is quite small, one could think that you would need a high-resolution display to determine the visual acuity. However, the Fresnel lenses of the HTC Vive manipulate the focal length, so that the vergence that is used to perceive virtual distance, is resembling the real world (21). In general, the HTC Vive has been found to be quite accurate in virtual distance projection (24). Keep in mind that the facerest of the HMD obstructs any additional lighting so use a dim-lit setting for testing visual acuity. Second, VR requires stereopsis for an undistorted perception. Therefore, a stereo acuity test should be performed before the session. For a quick and reliable assessment of stereopsis, the modified Random Dot Butterfly stereo test is a good option (for instructions see Chopin et al., 13).

### Soft- and Hardware

#### Experimental Routine

If the participant is eligible for participation, you should set the mandatory software. First, open the Pupil Core app and make sure that both eye cameras are displayed. You can define a threshold for low confidence. The standard setting is < 0.6; this threshold does influence the results of your calibration. If you would like to add some redundancy to match the Pupil Labs recordings with the Unity event file, you can annotate the *user info* with the participant’s code.

Although the technical specifications of the eye tracker list a sampling rate of 200Hz, the sampling rate should be checked before you continue with initializing the Unity application. Especially since the desired sampling rate is not saved necessarily, when the software is closed, but reset to the default setting for HMDs (currently 120Hz). Changing the sample rate is possible in the settings (cog symbol) in each eye camera window separately.

Second, open the Unity application, to avoid warning messages and to be certain, that the respective keys, triggering events from the *Gaze Tracker* component in the Pupil Core app, are also logged in the Unity event file. Moreover, the displayed Unity application should be indicating that the *Request Controller* uses the correct port, i.e. display “Connected”. For an easier handling of the software, using either a split screen or multiple screens for monitoring the eye cameras and the Unity environment simultaneously is recommended.

#### Fitting the HMD

After checking all necessary software prerequisites, continue with fitting the HMD. Adjust the head straps in the sagittal and the transversal axis according to the head circumference. Furthermore, the HTC Vive allows adjusting the IPD between 60.6 and 74.8 mm via a small wheel on the right side of the HMD. The IPD is measurable with a ruler that comes with the HTC Vive and should be individually fine-tuned. Moreover, the eye relief can be adjusted in the range from 13 to 24 mm, but the optimal distance is not easy to determine. Thus, the participant should assess all calibration markers as sharp when they are displayed with the preview function (keycode P when preview is enabled). In case the markers appear blurry the eye relief should be adjusted and the HMD should be fit again. The calibration will fail when the HMD is not properly fit. A proper fit can be assessed from the experimenter’s point of view through the eye camera windows, in which both eyes should be completely visible and centered.

As soon as the participant is wearing the HMD correctly, start the recording of the session to avoid the loss of information. The recording should be started inside the Unity application via the *Recording Controller* to avoid differences in time points, between Pupil Labs time and Unix epoc system time issued by Unity.

#### Calibration

**Figure 7:**
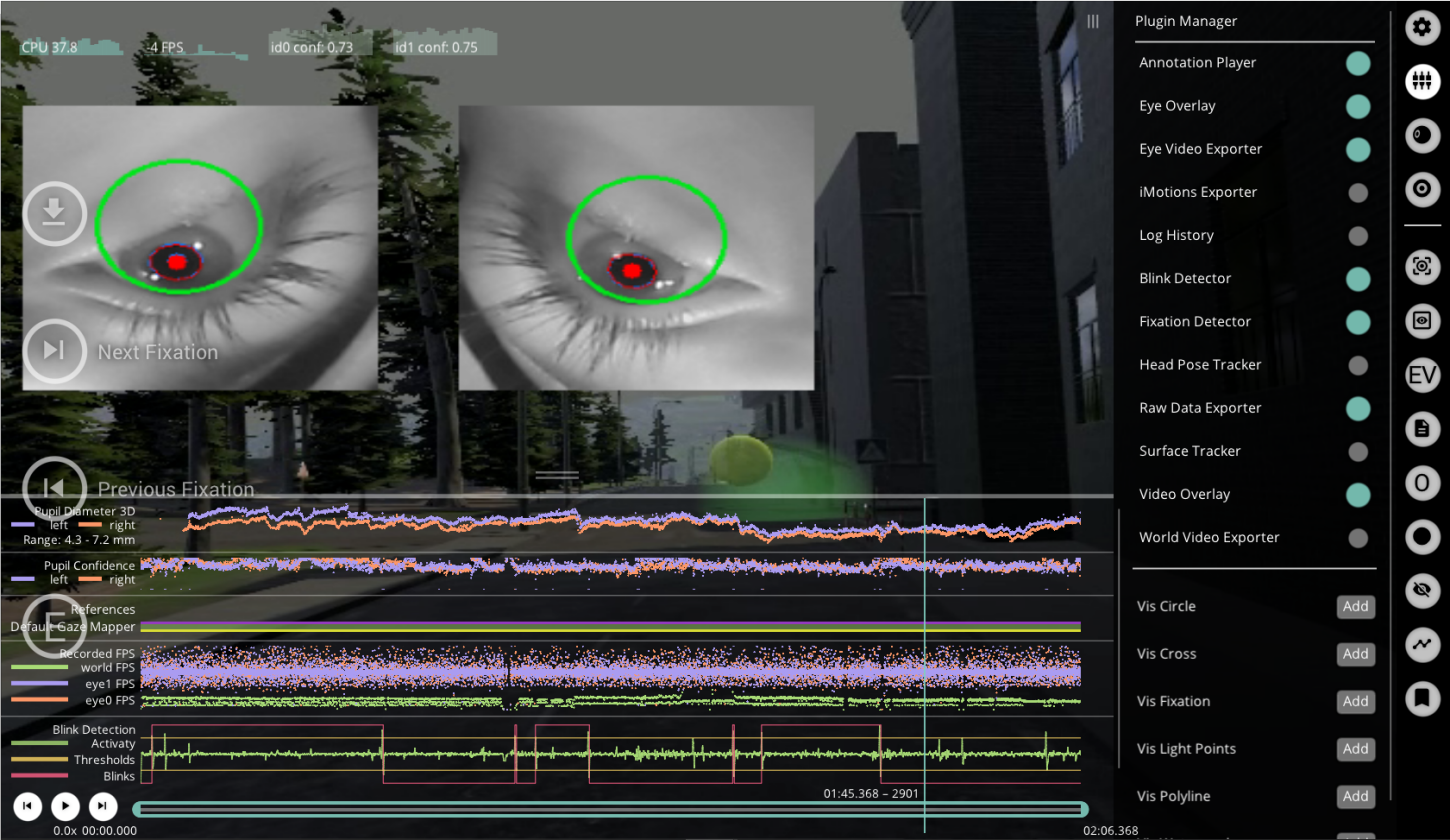
Graphic View of the Pupil Core App Pupil Player Note. The camera images of the eye cameras are displayed including the 3D eye model (green circles) and pupil detection (red circles) in the top section. In the background, the world video is visible. A variety of preprocessing functionalities is selected and the frequencies of the occurrences of eye events are shown underneath the screencast of the world video. On the right side, selectable and selected GUI elements for preprocessing can be found.

To provide a good basis for the Pupil Labs 3D pupil detection algorithm, instruct the participant to circle with their eyes. The Pupil Core app will start to adjust the 3D eye model that is represented by a green circle. When accurately recognized, it will match the eyeball. Moreover, there should be a red circle around the pupil with a red dot in the center (see Figure 7^2^). To reduce redundancy for other best practices, which are not hardware specific, the reader is referred to the Pupil Labs documentation (51). Before hitting C on the keyboard to start the calibration procedure, instruct the participant to reduce blinking while fixating on the targets during the calibration.

After the calibration, the amount of data below the predefined confidence threshold will be displayed in the console of the Pupil Core app. For comparable settings, valid trials included only data sets with between 70 and 80% of the data over the confidence threshold (52; 64). In case more than 30% of the data is missing, or whatever threshold you have predefined, reset the eye model(s) manually in the settings of the Pupil Core app you are using and restart the calibration.

## Accessing Raw Data

After terminating the VR application, you will find a folder with a running three-digit label in the path you set for saving the eye tracking recordings. The folder includes the info.player JSON file containing some metadata such as software and system version. In case it is used, the *user_info* table will include the participant’s code. Moreover, for troubleshooting and visualization, as well as offline pupil detection, the videos of the eye camera(s), and if a screencast is applied, the world video, can be found in this folder.

Additionally, the folder contains a variety of npy-files, which are effortlessly conveyable to readable csv-files via the Pupil Player. Hence, open the Pupil Player. Drag and drop the folder into the Pupil Player window. Once processed, the Pupil Player will display a window with a variety of selectable preprocessing functionalities on the right side (see Figure 7). Two raw data csv-files can be accessed in the plugin manager: the pupil positions and the gaze positions, moreover a text document with information about the meaning of the columns in the respective csv-files. Once selected, an additional GUI element with a progress bar will be displayed. After the progress bar is full, you can export the raw data by pressing E.

## Preprocessing

### Pupil Player

The Pupil Player not only exports raw data but also includes functionalities that can be used to preprocess the data before exporting them in csv-format: For instance, the dispersion-duration-based fixation detector. If you use a dispersion-duration based-fixation detector, apply an informed choice for the thresholds, as they affect the classification of the events (2). The same logic applies to the blink detector that allows setting the threshold for confidence, in onset and offset, as well as the selected length. As mentioned in the previous section, respective csv-files are exported by pressing E.

### Sampling Frequency

When visually inspecting the raw data, the sampling frequency will be around the selected sampling rate, but slightly varying over time and from eye to eye (for the binocular setup). This has been explained by competing processes that are run simultaneously on the computer (16). Moreover, in case binocular data are estimated with low confidence (i.e. < 0.6), data will be mapped monocular. It follows that there will be empty cells for certain timestamps which are not blinks. Especially for velocity-based algorithms sampling rates should be uniform (17).

There are two options for handling these special cases. The first one is direct processing which considers the irregularities by using the actual intervals between timestamps instead of the constant time. For algorithms that are depending on averaging over time points, e.g. for smooth pursuit eye movements, or over gaze positions for a cyclopean eye, e.g. visualization of monocular stimuli (18), this option cannot be suggested. The second option reconstructs the signal and allows a fixed sampling rate through interpolation. Each interpolation contains a different assumption about the data, which should be considered. Pupil Labs data has been linearly interpolated (39), but also advanced methods like piecewise cubic hermite interpolating polynomials (17) have been applied.

### Velocity Information Based on 3D-Gaze Points

Many research questions require the extraction of the gaze velocity information, based on the 3D-gaze points, which are available after the raw data (gaze position) export, for example when studying smooth pursuit eye movements. The depth of the gaze point is a derivative of the vergence that is provided via the gaze point 3D z-coordinate. For further information see the Pupil Tutorials page on GitHub (50). Even if you are not familiar with Python, you can apply the logic of the algorithm to any programming language you would like, e.g. in R.

### Merging Data via Timestamps

To match the system time in the Unix epoc and the Pupil Labs arbitrary timestamps you can look at the recording info.csv file that is included in the recordings. Set the first entry of the Pupil Labs timestamp to zero by subtracting all entries from the minimum and adding the system time to these timestamps. Next, the data from Unity and Pupil Labs can be merged. The usage of the nearest function is recommended, especially as sampling frequencies are likely to be different (maximum refresh rate of the HTC Vive 90Hz vs. sampling frequency Pupil Labs eye tracker 200Hz), and as mentioned previously sampling frequency might sometimes be volatile.

## Maintenance

**Figure 8:**
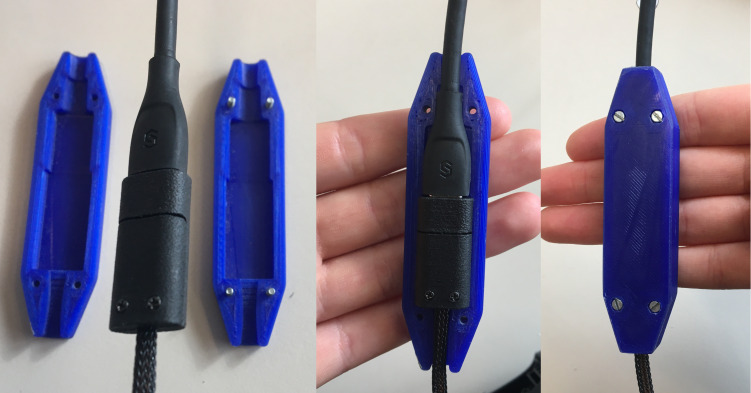
Assembly of the custom-built 3D-Printed Cable Protector Note. The 3D-printed cable protector is assembled by screwing the two molds together and it prevents the cable from damage due to rotational movement.

When combining eye tracking and VR, you might want to allow physical movement, or at least head rotation. Not only slippage (see the Calibration section) but also cable damage due to head movement might become problematic. Even though the flexible printed boards are protected against rupture with a flexible mesh tube, the rotational head movement during the VR exposure creates shear conditions. As a result, the flexible printed circuit boards are clinched and signals are lost, sometimes resulting in irreversible complete cable failure. Therefore, it is recommended that a 3D-printed cable protector is added to protect against damage caused by rotational movement (see Figure 8). The protector was custom-built using SolidWorks. First, a solid CAD model was created, for accessing the CAD model the reader is referred to the Electronic Supplementary Material section. Then, the 3D printing manufacturing parameters were set to 0.3 mm. After manufacturing, the molds were machined. The 3D-printed cable protector consists of two molds that can be screwed together. It can be slid under the cable management lug of the headband. This setup protects the delicate connection between the flexible printed circuit boards and the USB-C connector.

When the HMD is not in use for an extended period, cover the lenses and the eye tracker with a piece of cloth to prevent them from getting stretched or dusty. Moreover, you should protect the Fresnel lenses from direct sunlight, because the lenses act like burning glass, which can inflict heat damage to the display.

In contrast, when the eye tracker is intensely used the eye cameras might get greasy. This will become apparent in blurry images of the eye cameras displayed in the Pupil Core apps. To degrease and clean them, you can use lint-free alcohol wipes; there is no need to dismount the facerest.

## Discussion

This paper describes good practices and precautions when dealing with the Pupil Labs eye tracking add-on for the HTC Vive. The goal was to address low-threshold first steps and raise awareness for nifty prerequisites that might help researchers who want to get started with eye tracking in VR. The recommendations can be assessed as a blueprint, especially for novices.

Furthermore, the paper can be seen as a contribution in the context of open science. For eye tracking in VR recommendations are needed, not only because they are not established yet, but also because the used configuration should be made transparent. As there are many degrees of freedom for VR applications, as well as the definition of eye events, and equally valid solutions co-exist, this information should be part of the reporting.

Particularly concerning custom development, preprocessing, visualizations and analyses the possibilities for adaptations are diverse. Therefore, to achieve transparency and reproducibility with more customizability more effort into reporting is needed. Compared to proprietary solutions more information can and should be provided. Transparent reporting has implications for study design, evaluation, and reproducibility; thus existing guidelines should be considered (see Holmqvist et al., 23)

In contrast to competing companies, which are putting proprietary software for accessing the eye tracker at their disposal, the Pupil Core apps can be found on GitHub. This is an advantage in the context of open science and no license subscription is required for using the software. Additionally, it allows modifying the software according to the respective needs. GitHub repositories even enable participating in the development of software and sharing ideas for changes by submitting pull requests. Contributions of the community make the software sustainable, because users are neither depending on a valid subscription nor the continued support of the license.

Nevertheless, it is questionable whether a researcher needs to be involved in the software development process. First of all, the likelihood of erroneous code and the time required to create a custom application can be judged as inefficient. This is especially true if someone has to make a trade-off between speed and accuracy since the project durations are not unlimited − a statement that can be applied to all open science efforts. Especially for optimizing the procedures a high level of expertise is necessary, which can be hard to acquire (58). Moreover, using "plug-and-play" solutions is not only more convenient and faster, but also distributes the responsibility for the correctness of the procedures between the researcher and the manufacturer, in line with the principle: “Ignorance is bliss”. However, I consider these arguments weak compared to the knowledge gained, the achievable sustainability, and the empowerment of the researcher.

For a faster and easier click-through analysis, proprietary analysis software is available. Thus, some preprocessing, analysis, and visualization can be outsourced. For this purpose, the Pupil Player contains an export function for iMotions data format, but other analysis software like BlickShift analytics is also able to read the Pupil Labs exports in csv-format, facilitating the preprocessing and visualization. However, the subscription to this software is costly and should be judged in the context of the respective research question. Consequently, it might be worthwhile to take a look at Pupil Labs Tutorials on GitHub with the research question in mind. This might empower the researcher to develop the required algorithms by oneself, thereby enabling one to control and manipulate the code according to the needs, but also get a better understanding of eye tracking in general.

I would like to stress that, some of the suggestions are general-purpose: Not only when you are using another Vive product (Vive Pro or Vive Cosmos) that is compatible with the Pupil Labs add-on, but also when using a different eye tracker. Furthermore, even with different eye tracking devices in VR, there is the need for calibration, to sample a Unity event file, the consideration for minimum requirements for participants, handling, and maintaining the hardware. Additionally, actuating the eye tracker from inside Unity is similar to the integration of other build-in eye trackers for HMD: For instance, the integration of the HTC Vive integrated Tobii eye tracker inside Unity via the Tobii XR SDK (60). This SDK allows for loading an initialization script from inside Unity. Accordingly, only the Unity application needs to be started and all default settings for the eye tracking software will be initialized simultaneously. This might inspire a reader who is adept in programming to write a similar initialization script for the Pupil Core apps, which would facilitate the setup process and reduces the likelihood of errors.

Moreover, to take advantage of the combination of eye tracking and VR researchers should borrow from best practices for other remote and head-mounted systems (22), but keep the specialties of VR in mind. Eye tracking in VR can be thought of as a hybrid form in terms of controllability and constraint. Since VR allows the use of an interactive and less restricted setup, e.g. allowing head movement, while having high controllability of the visual stimuli.

Compared to head-mounted eye trackers for the physical world the analysis of previously defined AOI via the *Gaze Tracker* component and some C# code is rather simple. Additionally, if the perspective is important, it is advisable to constrain the y-axis in Unity coordinates as mentioned in the section on Software Integration for a height-independent VR perception, which is of course impossible for eye tracking in the physical world. Despite the unnaturalness, it is an advantage especially for studying easily confounded eye events like pupillometry (15).

As movement is not only possible physically but also virtually via controllers, controlling movement can become cumbersome. Thus, it is advisable if the laboratory allows room-scale VR to take a pass on controller input for virtual movement, which as a side benefit increases the perceived comfort (56). Nevertheless, to avoid slippage the degrees of freedom for physical head movement should be restrained. A suitable compromise can be allowing only physical rotational head movement and using the controller input for translational movement or applying teleportation.

The Pupil Labs head-mounted eye tracker, which is using the same algorithms for the 3D eye model, as the add-on for the HTC Vive, was found to be prone to errors due to talking and facial expressions (41). Therefore, a facial add-on could control for these artifacts in future setups (67). Additionally, to make the most of the combination of eye tracking and VR wire-less alternatives should be pursued while aiming for comparable low signal processing latencies as cable-based solutions. Until then, providing longer connector cables can be a makeshift solution, analogous to the discontinued backpack computers.

Despite the analogies, keep the differences between the virtual and the physical world in mind. As stated previously, achievable controllability is even higher than in the physical world. Nevertheless, spatial and temporal accuracy and precision are not comparable to modern remote eye trackers (17). Moreover, promoted accuracy and precision might not be achievable if conditions are suboptimal. Therefore, it might be worthwhile to test accuracy and precision in the custom application used, e.g. via the open source suite GazeMetrics (1). However, even if the accuracy and precision are sufficient, answering some research questions is still limited with this technology. For example, whenever there is a need for averaging over timespans because the sampling frequency is volatile. Furthermore, whenever the AOIs are small, the resolution not only of the eye cameras but also of the HMD is limited. This ongoing resolution problem is called the screen door effect − a mesh-like optical effect that resembles a screen door (40).

In general, the Pupil Labs eye tracking add-on for the HTC Vive can be assessed as an affordable and transparent solution with many degrees of freedom. Thus, the raw data, the preprocessing, and the analysis of the data are customizable. With a little programming knowledge, you can integrate the necessary plugin in Unity and write your own routines to visualize and analyze the eye tracking data in VR. As expected for open source code software, a lot of useful information can be found online. For individual questions, support is also available cost-neutral on the Pupil Labs Discord channel, in which the text channels can be browsed via the search function, as well as new questions be raised. Moreover, you can even become a part of the software development via GitHub.

To conclude, open source solutions like the one provided by Pupil Labs with very active community support and helpful information on GitHub should be pursued to enable flexible, accessible, transparent, and sustainable eye tracking in VR.

### Ethics and Conflict of Interest

The author(s) declare(s) that the contents of the article are in agreement with the ethics described in http://biblio.unibe.ch/portale/elibrary/BOP/jemr/ethics.html and that there is no conflict of interest regarding the publication of this paper.

### Acknowledgements

I wish to thank my two colleagues of the Technische Universität Dresden: Sebastian Pannasch for his time, support, and patience to copyedit this article and Gernot Pascher for sharing the CAD model for the 3D-printed cable protector provided in the Electronic Supplementary Material.

The Article Processing Charges (APC) were funded by the joint publication funds of the TU Dresden, including Carl Gustav Carus Faculty of Medicine, and the SLUB Dresden as well as the Open Access Publication Funding of the DFG.

## Footnotes

1 In case you are using the newer version, the HTC Vive Pro, there is a detailed instruction for dismounting the HMD’s face-rest online (43).

2 To avoid confusion: the illustration shows the 3D eye model, neither in Pupil Capture nor in Pupil Service, but based on post-hoc pupil detection in Pupil Player.
